# Prolonged urinary leakage in the postoperative period of renal hydatic cyst treatment with oral desmopressin: a case report

**DOI:** 10.1186/1752-1947-6-339

**Published:** 2012-10-05

**Authors:** Cavit Ceylan, Özkan Baytok, Serkan Doğan, Metin Yığman

**Affiliations:** 1 Clinic of Urology, Türkiye Yüksek Ihtisas Training and Research Hospital, Ankara, Turkey

**Keywords:** Desmopressin treatment, Hydatic cyst, Renal leakage

## Abstract

**Introduction:**

Prolonged extravasation after renal and ureter surgeries is a bothersome situation for both the patient and the doctor. It is usually related to the suture line not being watertight. The contact between urine and the edges of the wound also delay healing of the wound. In this situation, the first thing to do is to break the contact between the wound and the urine by inserting an adequate stent. Sometimes, this process is not enough. We approached this problem with a different treatment method for a case involving prolonged drainage.

**Case presentation:**

A 52-year-old Caucasian woman who presented at our clinic with right flank pain was operated on due to a renal hydatic cyst, and cyst removal was performed. On follow-up, prolonged urinary leakage was observed and a desmopressin treatment was started on the patient. Drainage was greatly reduced after desmopressin was started and there was no drainage on the fifth day.

**Conclusion:**

Prolonged extravasation is a bothersome situation and there can be many reasons for this. Whenever traditional approaches are not enough, oral desmopressin therapy can be started reliably if there are no contraindications for the patient. Eventually, contact between urine and the suture site will cease and therefore the fever and healing time will be shortened.

## Introduction

Urine leakage and urinoma occur as a result of the disruption of the urinary collecting system at any level from the calyx to the urethra. Persistent urine leakage is frequently seen following iatrogenic injuries such as open and/or laparoscopic renal surgery and percutaneous nephrolithotripsy [1-3]. Dealing with urine leakage generally involves some applications, such as catheter drainage, ureteral stenting and percutaneous nephrostomy placement with open repair versus nephrectomy which is reserved for persistent leakage [1]. However, to the best of our knowledge, the effectiveness of medical therapy in urinary leakage has not been mentioned previously. We claimed that reduction in urinary output can accelerate improvement of urinary leakage. In an effort to test this hypothesis, we used desmopressin, which has antidiuretic effects and can decrease urinary output. Thus, we report our initial experience regarding the effect of desmopressin in reduction of urinary leakage duration following urological surgeries.

## Case presentation

**Figure 1:**
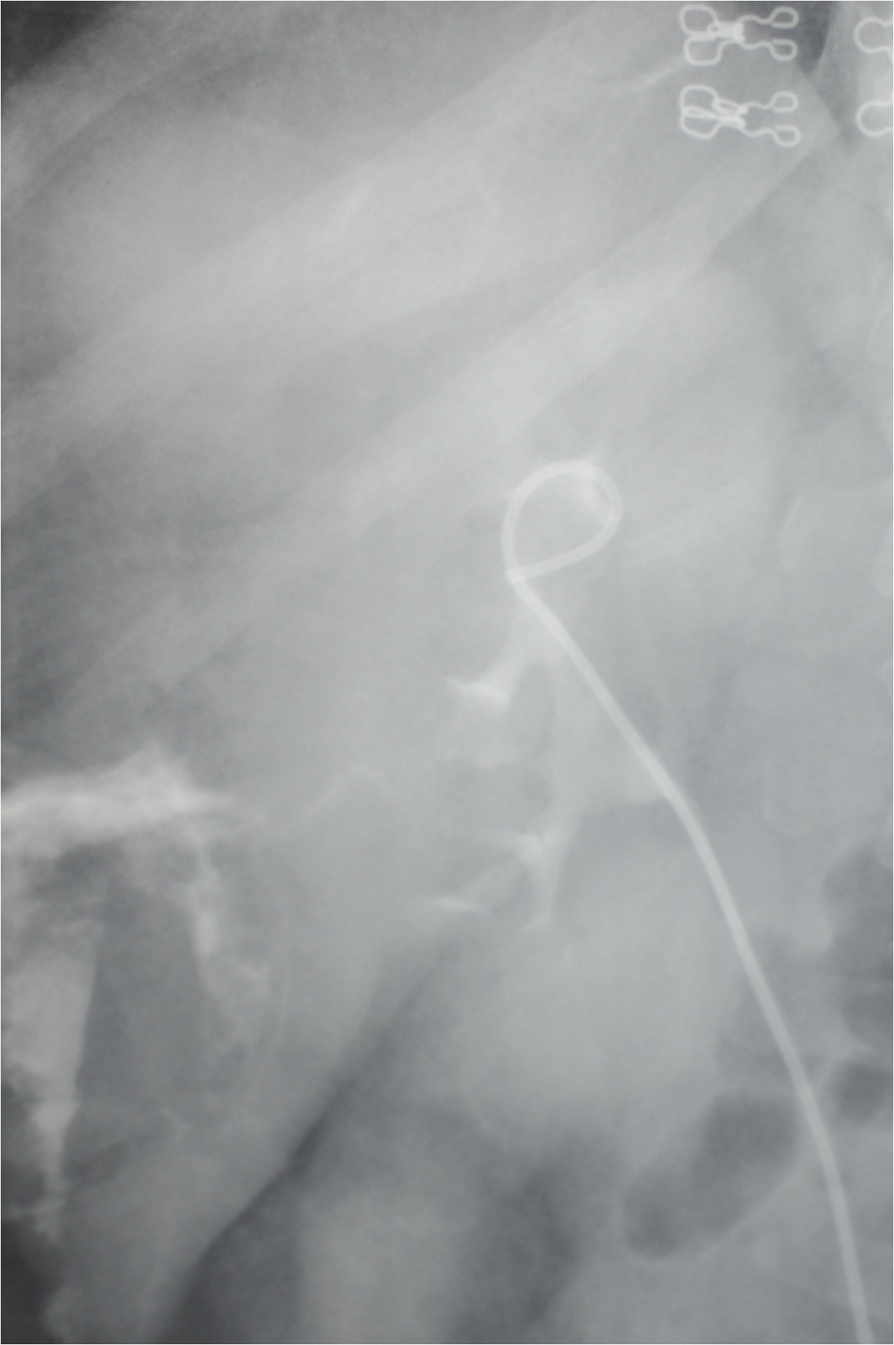
A view of urinary leakage in intravenous pyelography.

**Figure 2:**
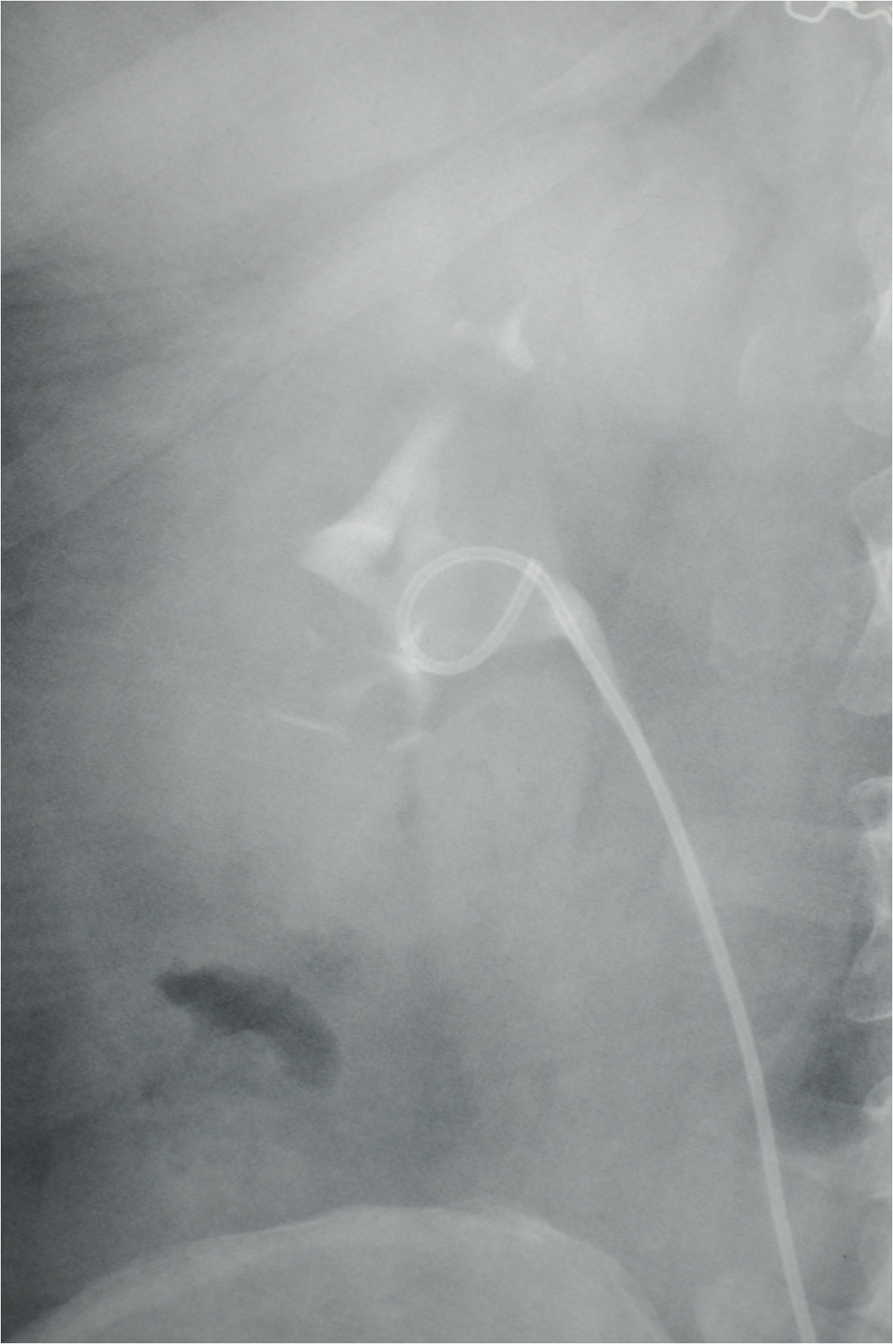
A view of the improved urinary leakage in intravenous pyelography after oral desmopressin therapy.

In the examination of a 52-year-old Caucasian woman with a 2-year history of right flank pain and lower urinary tract symptoms, a cystic mass was observed in her right kidney in ultrasonography. The cystic mass was diagnosed as a renal hydatic cyst in computed tomography. The patient was hospitalized with the diagnosis of renal hydatic cyst and no abnormality was found in laboratory and urine testing. No laboratory analysis was performed for a hydatic cyst; however, a renal hydatic cyst was diagnosed as a result of radiological findings. The patient’s history revealed no close relationship with animals. It was learned that the patient received medical treatment because of cerebrovascular disease and that she had no neurologic deficit for the time being. She had only taken medical treatment due to collagen tissue illness for 15 years. Subsequently, she was accepted to our clinic for surgery and open complex cyst removal was done under general anesthesia. The kidney was sutured watertight. However, in the postoperative period, a ureteral double j stent was placed because the drainage did not stop in the following 3-week duration. Because drainage of 250cc daily continued without any decrease the week following the double j stent placement, intravenous pyelography (IVP) was applied and urinary leakage was found (Figure 1). Urinary drainage was more prominent at night than during the day (250 versus 50cc). Subsequently, oral desmopressin was started at a dosage of 0.2mg/day in order to decrease the nocturnal urinary drainage; no abnormal situation was observed in serum electrolytes (specifically hyponatremia). The patient’s drainage decreased dramatically to a daily total of 50cc and she was discharged on the fifth day of the medical treatment after the drainage had stopped. After 1 week when the patient came for follow-up, the desmopressin treatment was ended. IVP was applied 3 weeks after the patient was discharged, and a urogram showed that the two kidneys were active and the double j stent was removed because no urinary leakage was observed (Figure 2).

## Discussion

Desmopressin is a drug which has been used as a synthetic arginine vasopressin analog for 40 years. This drug has been proved to be effective for the treatment of nocturnal enuresis, central diabetes insipidus and some coagulopathies. The antidiuresis induced by desmopressin is more potent than that of arginine vasopressin, resulting in an increased urine osmolality and decreased urine output. Several investigators have demonstrated significant decreases in nocturnal diuresis and subsequent improvements in sleep [4-7]. In the study of Razzaghi et al. nasal desmopressin (Minirin®) was applied to 7 out of 15 patients and 8 of them were observed without taking any treatment. Urinary leakage duration reduced significantly in the group who had desmopressin (Minirin®) treatment. They observed a dramatic decrease in urine leakage in the group to whom Minirin® was given. They found this result to be statistically significant [8]. Furthermore, Çimentepe *et al*. reported on a patient whose prolonged urinary leakage following percutaneous nephrolithotomy, which had not stopped although a double pig-tail stent had been inserted, suddenly came to an end with peroral desmopressin treatment. They showed that treatment with oral desmopressin could reduce urinary leakage [9]. In our case, the urinary leakage continued although we inserted a double j stent, but it reduced dramatically during the week following the commencement of oral desmopressin treatment and no complication was observed in the patient. Our study showed that desmopressin can reduce the duration of urinary leakage in these patients.

The use of desmopressin may improve the beneficial effect of other procedures such as catheter drainage, ureteral stenting, and percutaneous nephrostomy placement for incisional urinary leakage. Thus, we have come to the conclusion that patients with prolonged urinary leakage, who do not respond to surgical urinary drainage after renal surgeries, can benefit from desmopressin. We argue that desmopressin medical treatment can be considered an alternative in clinics with many centers and patients and in prolonged clinical studies.

## Conclusion

Desmopressin treatment should be considered a safe alternative treatment in prolonged urinary leakage before it becomes chronic, which is an annoying complication in the postoperative period. Desmopressin can be applied orally and it presented no complication in recent blood tests.

## Consent

Written informed consent was obtained from the patient for publication of this case report and accompanying images. A copy of the written consent is available for review by the Editor-in-Chief of this journal.

## Competing interests

The authors declare that they have no competing interests.

## Authors’ contributions

OB and SD analyzed and interpreted the patient data regarding the renal hydatic cyst. CC and MY performed the surgery of the renal hydatic cyst and began desmopressin treatment. CC was a major contributor in writing the manuscript. All authors read and approved the final manuscript.
